# Laponite^®^-Based Smart Hydrogels for Sustained Topical Delivery of Silver Sulfadiazine: A Strategy for the Treatment of Contaminated or Biofilm-Forming Wounds

**DOI:** 10.3390/pharmaceutics17091234

**Published:** 2025-09-22

**Authors:** Jonas Lira do Nascimento, Michely Conceição Viana da Costa, Leticia Farias de Macêdo, Luiz Henrique Chaves de Macêdo, Ricardo Olímpio de Moura, Tomás Jeferson Alves de Mélo, Wilma Raianny Vieira da Rocha, Ana Cristina Figueiredo de Melo Costa, José Lamartine Soares-Sobrinho, Dayanne Tomaz Casimiro da Silva

**Affiliations:** 1 Pharmaceutical Sciences Postgraduate Program, Paraiba State University, UEPB, Campina Grande 58429-500, PB, Brazil; jonas.lira.n@gmail.com (J.L.d.N.); ricardo.olimpiodemoura@servidor.uepb.edu.br (R.O.d.M.); wilmaraianny@gmail.com (W.R.V.d.R.); 2 Pharmacy Department, Paraiba State University, UEPB, Campina Grande 58429-500, PB, Brazil; michely.costa@aluno.uepb.edu.br (M.C.V.d.C.); henriquechaves2000@hotmail.com (L.H.C.d.M.); 3 Pharmaceutical Sciences Postgraduate Program, Federal University of Pernambuco, UFPE, Recife 50740-521, PE, Brazil; leticiafariasm9@gmail.com (L.F.d.M.); jose.ssobrinho@ufpe.br (J.L.S.-S.); 4 Postgraduate Program in Materials Science and Engineering (PPGCEMat), Federal University of Campina Grande (UFCG), Campina Grande 58429-900, PB, Brazil; tomas.jeferson@professor.ufcg.edu.br (T.J.A.d.M.); ana.figueiredo@professor.ufcg.edu.br (A.C.F.d.M.C.)

**Keywords:** drug delivery system, topical formulation, hydrogels, biofilm inhibition

## Abstract

**Background/Objectives**: Silver sulfadiazine (AgSD) is widely used in the topical treatment of burns and infected wounds, but its conventional formulations present drawbacks such as poor water solubility, the need for multiple daily applications, and patient discomfort. To overcome these limitations, this study aimed to develop and evaluate Laponite^®^ (LAP)-based hydrogels loaded with AgSD for controlled release and enhanced antimicrobial and antibiofilm efficacy, offering a promising alternative for the treatment of contaminated or biofilm-forming wounds. **Methods**: Laponite^®^-based hydrogels containing 1% and 1.2% AgSD (LAP@AgSD) were prepared using a one-pot method. The formulations were characterized rheologically, thermally, and structurally. In vitro drug release was assessed using Franz diffusion cells, and mathematical modeling was applied to determine release kinetics. Antibacterial and antibiofilm activities were evaluated against *Staphylococcus aureus*, *Escherichia coli*, and *Pseudomonas aeruginosa* using standardized microbiological methods. **Results**: LAP@AgSD hydrogels exhibited pseudoplastic behavior, high structural integrity, and enhanced thermal stability. In vitro release assays revealed a sustained release profile, best fitted by the Weibull model, indicating diffusion-controlled mechanisms. Antibacterial assays demonstrated concentration-dependent activity, with LAP@AgSD 1.2% showing superior efficacy over LAP@AgSD 1% and comparable performance to the commercial silver sulfadiazine cream (CC-AgSD). Biofilm inhibition was significant for all formulations, with CC-AgSD 1% exhibiting the highest immediate activity, while LAP@AgSD 1.2% provided sustained antibiofilm potential. **Conclusions**: LAP-based hydrogels are promising smart delivery systems for AgSD, combining mechanical robustness, controlled drug release, and effective antibacterial and antibiofilm activities. These findings support their potential use in topical therapies for infected and chronic wounds, particularly where biofilm formation is a challenge.

## 1. Introduction

Skin lesions have always been a common occurrence in human daily life, representing a problem caused by different etiologies such as burns, cracks, incisions, and infections, which characterizes them as a global public health issue [1]. Due to their exposed and vulnerable nature, they are susceptible to a range of risks, including contamination by pathogenic microorganisms, which can worsen their pathophysiological condition and delay the healing process. Therefore, the effective treatment of these lesions should not only promote patient recovery but also play a key role in preventing infections and subsequent complications, as they represent a significant public health concern [2,3,4].

In this context, the protocol for the prevention and topical treatment of wounds at risk of generalized infection, particularly burns and exudative ulcers, has employed 1% silver sulfadiazine (AgSD) cream, which exhibits both bactericidal and bacteriostatic activity against Gram-negative bacteria. The World Health Organization (WHO) [5] recommends its use for the topical treatment of partial- and full-thickness burns to prevent bacterial infection. The Wound Healing Society (WHS) guidelines [6] also recommend its use for burns or wounds with a high risk of bacterial colonization, especially those caused by *Pseudomonas aeruginosa*. Furthermore, it is indicated for wounds suspected of biofilm formation, such as chronic wounds that do not respond to standard treatments, with persistent exudate and necrotic tissue [7].

However, AgSD presents certain limitations due to its low water solubility, which compromises its activity, as the antimicrobial effect depends on the dissociation of sulfadiazine (which inhibits folic acid synthesis necessary for bacterial reproduction) and the release of silver ions (Ag^+^), which cause protein denaturation and disruption of the bacterial cell membrane [8,9]. Moreover, depending on the pathophysiology and clinical status of the lesion, two to four applications per day may be required. This can lead to significant patient discomfort, as the currently available formulation leaves a residue upon drying that must be removed during wound cleansing, often causing pain. This discomfort may reduce treatment adherence, prolong the healing process, or even lead to treatment failure [10,11,12].

From this perspective, the selection of versatile and multifunctional drug carriers has been investigated to improve physicochemical properties and enable controlled drug release. Various materials are available for the formation of nanocarriers [13], including polymers [8], lipids [9], surfactants [14], and inorganic materials [11,15]. One example of such an inorganic material used as a drug carrier is clay minerals, such as Laponite^®^ (LAP) [16,17,18], a synthetic 2:1 phyllosilicate, which consists of an octahedral layer sandwiched between two tetrahedral layers. Its empirical chemical formula is Na_0.7_[(Si_8_Mg_5.5_Li_0.3_)O_20_(OH)_4_], and it forms nanodiscs measuring 20–50 nm in diameter and 1–2 nm in thickness. Due to isomorphic substitution in its structure, lower-valence cations such as lithium (Li^+^) replace higher-valence cations such as magnesium (Mg^2+^), generating a net negative charge that is counterbalanced by exchangeable cations like sodium (Na^+^) located in the interlayer space, where entrapment and stabilization of substances can occur. Furthermore, when dispersed in aqueous solution, the nanodiscs can interact via electrostatic attraction (edge–face) or repulsion (face–face, edge–edge), leading to the reorganization of lamellae into a three-dimensional gel structure [18,19,20,21,22].

Thus, due to the versatility of these clay minerals, some studies have employed these compounds as vehicles for the controlled release of drugs, especially for the treatment of skin injuries. Pacelli et al. (2016) [23], for example, used Laponite^®^ (LAP) in combination with gellan gum (functionalized with methacrylate groups) to form a biocompatible injectable hydrogel designed for the release of ofloxacin in the treatment of skin wounds, particularly burns. In this formulation, LAP acted as a crosslinker between the rigid polymer chains, providing both strength and flexibility, thereby enabling the creation of stable hydrogels and representing a strategy to control drug release over time as an intelligent device embedded with therapeutic agents. More recently, Zhou et al. (2024) [24] developed an injectable hydrogel composed of Laponite^®^ and lactoferrin, incorporated with eugenol for the healing of chronic skin wounds infected by methicillin-resistant *Staphylococcus aureus* (MRSA), offering antioxidant, hemostatic, and antibacterial properties. Despite these promising advances, studies incorporating the clinical standard silver sulfadiazine (AgSD) into Laponite^®^-based hydrogels are still scarce. Most reported systems have focused on antibiotics [25,26,27] or natural compounds [28,29,30,31], thereby leaving an important gap for innovative strategies that can overcome the well-known limitations of AgSD in wound therapy.

Thus, considering the limitations of conventional silver sulfadiazine (AgSD) formulations and the clinical challenges associated with AgSD-based pharmacotherapy for skin lesions, along with the potential of novel material combinations, this study aims to develop a hydrogel based on Laponite^®^ (LAP) and AgSD (LAP@AgSD), prepared using a one-pot mixing method for the controlled release of AgSD. The study further aims to evaluate its antimicrobial and antibiofilm efficacy, with the ultimate goal of providing new insights for the design and clinical translation of wound dressings for infected skin injuries.

## 2. Materials and Methods

### 2.1. Materials and Reagents

Silver sulfadiazine (AgSD, 99%) was obtained from Fagron (Rotterdam, The Netherlands), Laponite^®^ (LAP) RD from Colormix (Hong Kong, China), ammonium hydroxide (NH_4_OH, 38%) and citric acid from Dinâmica (Indaiatuba, São Paulo, Brazil), monobasic and dibasic phosphate from Nuclear, and the commercial silver sulfadiazine dermatological cream (CC-AgSD 1%) from Pratti Donaduzzi (Toledo, Paraná, Brazil). All materials were used as received, and all solutions employed in the experiments were prepared with deionized water (H_2_O).

### 2.2. Influence of Concentration and Stirring Time on LAP Hydrogel Formation Apparent Viscosity

To determine the optimal LAP hydrogel formulation for the incorporation of AgSD, the effects of LAP concentration and stirring time were systematically evaluated. Hydrogels were prepared at three different LAP concentrations (3.2%, 3.4%, and 3.8% *w/v*), dispersed in water (designated as LAP 3.2%, LAP 3.4%, and LAP 3.8%), homogenized by magnetic stirring (IKA Works INC., Wilmington, NC, USA), at 450 rpm, and subjected to three different stirring times (60, 120, and 720 min). Each formulation was prepared in a final volume of 10 mL at controlled room temperature (25 ± 2 °C) without light protection. After preparation, the hydrogels were transferred to sterile glass vials and stored at 4 °C until further use. The apparent viscosities of the formulations were determined using a Brookfield Viscolead One digital rotational viscometer (Fungilab^®^, Barcelona, Spain) equipped with a coaxial cone spindle R7, which was immersed in the samples. These concentrations and stirring times were selected based on previous studies by Suterio et al. (2022) [18], which showed that apparent viscosity decreases with increasing temperature and that higher concentrations lead to more pronounced phase transitions. Furthermore, Chen et al. (2022) [25] demonstrated that increasing the concentration of LAP in the gelling solution improves the thixotropic performance of the formulation [32]. All measurements were performed in triplicate for each formulation.

### 2.3. Preparation of LAP@AgSD Hydrogels Using the One-Pot Method

To evaluate the influence of varying AgSD concentrations within the safe range for topical use and their effect on formulation conditions, hydrogels were prepared containing AgSD at 1% and 1.2%. AgSD was dissolved in a 38% NH_4_OH solution, which was then added dropwise to the LAP 3.8% hydrogel under magnetic stirring at 450 rpm for 60 min. The pH of the formulation was subsequently adjusted with a 20% citric acid solution to reach values close to that of the skin (5.0 ± 0.6), as described by Suterio et al. (2022) [18]. All formulations were prepared in amber glassware to protect them from light, with a final volume of 10 mL under controlled room temperature (25 ± 2 °C). After preparation, the hydrogels were transferred to sterile glass vials and stored under refrigeration (4 °C) until further use.

### 2.4. Rheological Characterization of LAP@AgSD Hydrogels

The rheological characterization of LAP hydrogels containing 1% and 1.2% AgSD (LAP@AgSD 1% and LAP@AgSD 1.2%) was performed using a controlled-stress oscillatory rheometer (MCR 301, Anton Paar^®^, Graz, Austria), equipped with a parallel plate geometry (25 mm diameter, 1 mm gap), suitable for the analysis of semisolid samples. The tests were conducted under an inert nitrogen (N_2_) atmosphere at a controlled temperature of 25 ± 0.1 °C. To evaluate the linear viscoelastic behavior of the hydrogels, a frequency sweep test was performed over the range of 0.1–10 rad·s^−1^, with a constant strain within the linear viscoelastic region previously determined by a strain sweep test. During the assay, the storage modulus (G′), loss modulus (G″), damping factor (tan δ = G″/G′), and complex viscosity (η*) were recorded, allowing for the comparison of the structural performance and mechanical strength of the hydrogels across different formulations.

### 2.5. Thermal Characterization of LAP@AgSD Hydrogels

Thermogravimetric analysis (TGA) was performed using a DTG-60H thermobalance (Shimadzu^®^ Analytical and Measuring Instruments, Kyoto, Japan). The heating rate was set at 10 °C·min^−1^, and the temperature range extended up to 800 °C under a nitrogen atmosphere with a constant flow rate of 50 mL·min^−1^. For differential scanning calorimetry (DSC) analysis, a DSC-60 Plus instrument (Shimadzu^®^, Kyoto, Japan) was used. Samples of approximately 2.5 mg were placed in aluminum crucibles and subjected to controlled heating under a nitrogen atmosphere with a flow rate of 50 mL·min^−1^, ensuring an inert environment. The temperature range used for the analysis was 5–300 °C, with a heating rate of 10 °C·min^−1^.

### 2.6. Structural Characterization of LAP@AgSD Hydrogels

Structural characterization of the hydrogels was carried out using Fourier-transform infrared spectroscopy (FTIR) and zeta potential analysis. FTIR spectra of AgSD, LAP, the LAP hydrogel, and the LAP@AgSD hydrogels were obtained at room temperature using a Vertex 70 FT-IR spectrometer (Bruker, Billerica, MA, USA; model 660-IR). The spectra were recorded in the range of 4000 to 400 cm^−1^, with a resolution of 4 cm^−1^ and 32 scans. Additionally, the zeta potential of the samples was determined at pH 5.0 using a HORIBA Scientific nanoparticle analyzer (SZ-100 series, Kyoto, Japan). Zeta potential values above +30 mV or below −30 mV are considered indicative of stable colloidal systems, whereas values within −30 mV to +30 mV suggest instability, providing insight into the surface charge and colloidal stability of the formulations.

### 2.7. Morphological Characterization of LAP and LAP@AgSD Hydrogels by Transmission Electron Microscopy (TEM)

The particle agglomerates were analyzed using Transmission Electron Microscopy (TEM). A Tecnai G2 Spirit Twin microscope (FEI Company, Hillsboro, OR, USA) operating at 120 kV was employed. Samples of blank Laponite^®^ hydrogel and AgSD-loaded hydrogels (1.0% and 1.2%) were diluted in distilled water and deposited onto carbon-coated copper grids. The excess liquid was removed using filter paper, and the grids were allowed to dry at room temperature before analysis. Images were acquired in bright-field mode to visualize the morphology and organization of the clay lamellae, as well as the microstructural changes upon drug incorporation.

### 2.8. In Vitro Release in Franz Diffusion Cells Using a Synthetic Membrane

In vitro release was performed using a vertical Franz diffusion cell apparatus with six individual compartments, each with a diffusion area of 0.7359 cm^2^. Each cell consisted of a receptor compartment with an approximate volume of 7 mL and a donor compartment with an approximate volume of 3 mL. Artificial hydrophilic cellulose acetate membranes with a pore diameter of 0.45 µm (Millipore^®^, Barueri, Brazil) were used for sample diffusion. All cells were connected to an ultra-thermostatic bath maintained at 37 ± 0.5 °C and to a stirring system, with constant agitation at 100 rpm throughout the 24 h experiment. The receptor solution used was phosphate buffer (PBS, pH 7.4). The hydrophilic cellulose acetate membranes were placed on top of the receptor compartment. After assembling the Franz cells, the AgSD hydrogels and the commercial cream were directly placed into the donor compartment, and the system was then sealed. For each experiment, 100 mg of hydrogel was applied to the donor compartment, covering an effective membrane contact area of 0.7539 cm^2^. Samples from the receptor solution were collected at predetermined time intervals of 10, 30, 60, 120, 180, 360, 720, and 1440 min. The entire receptor solution was collected and immediately replaced with fresh PBS to maintain sink conditions. The cumulative amount of AgSD released through the membrane was calculated based on the area (µg/cm^2^), and the results were plotted as a function of time (Equation (1)). Quantification was performed using a UV–Vis spectrophotometer (UV-1800, Shimadzu^®^, Kyoto, Japan) at λ = 255 nm. All experiments were performed in sextuplicate.

(1) $$\%\;Release=\frac{Total\;amount\;released\;(µg/{cm}^{2})\times Diffusion\;area\;({cm}^{2})}{Medium\;concentration\;(mg/mL)\times Medium\;volume\;(mL)}\times 100$$

The release kinetics of AgSD were evaluated using five different theoretical mathematical models based on the in vitro transdermal drug release data [33,34,35]. These models included the zero-order model (µg/cm^2^ versus time), first-order model (log µg/cm^2^ versus time), Korsmeyer–Peppas model (log µg/cm^2^ versus log time), Peppas–Sahlin model (fraction of drug released versus time), and Weibull model (log[−ln(1 − fraction of drug released)] versus log time). The model that best described the drug release from the hydrogel was determined based on the correlation coefficient (r^2^), using the equations presented (Equations (2)–(6)):

Zero-order [36]

(2) $$Q\;=\;Q_{O}+k_{O}t$$

First-order [37]

(3) $$logC_{t}={logC}_{O}-k_{t}/2.303$$

Korsmeyer–Peppas [38]

(4) $$\frac{Mt}{M\infty }={kt}^{n}$$

Peppas–Sahlin [39]

(5) $$\frac{Mt}{M\infty }=k_{1}t^{m}+k_{2}t^{2m}$$

Weibull [40]

(6) $$F\left(t\right)=F_{max}.\left(1-exp\left[-\left(\frac{t-T_{i}}{\alpha }\right)\beta \right]\right)$$

where Q is the amount of drug released; Q_0_ is the initial amount of drug in the solution; k_0_ is the zero-order release constant; C_0_ is the initial drug concentration; and C_t_ is the drug concentration in the solution at time t. M_t_/M∞ is the fraction of drug released at time t, and k is the release rate constant. k_1_ is the Fickian diffusion constant; k_2_ is the matrix relaxation constant (non-Fickian diffusion); m is the release exponent associated with the release mechanism (geometric and physicochemical); t is time; F(t) is the amount or fraction of drug released at time t; F_max_ is the maximum release value; T_i_ is the lag time before the onset of release; a is the scale parameter; and β is the shape parameter.

### 2.9. Evaluation of Antibacterial Activity

#### 2.9.1. Preparation of the Bacterial Suspension and Inoculum Standardization

To determine the antimicrobial activity based on the minimum inhibitory concentration (MIC) of the AgSD hydrogels, microbial strains from the American Type Culture Collection (ATCC) were used: *Staphylococcus aureus* (ATCC 25923), *Pseudomonas aeruginosa* (ATCC 27853), and *Escherichia coli* (ATCC 25922). These strains were maintained in brain heart infusion broth (BHIB) (DIFCO^®^, Franklin Lakes, NJ, USA) supplemented with 20% (*v/v*) glycerol. The inoculum was standardized according to the guidelines of the Clinical Laboratory Standards Institute (CLSI) M07 [41], using a Mueller-Hinton broth (MHB, DIFCO^®^) cultures incubated for 24 h at 35 ± 2 °C. The inoculum was adjusted to match the 0.5 McFarland turbidity standard in MHB, and for the assays, the initial inocula were diluted to a final concentration ranging from 2.0 to 8.0 × 10^5^ CFU/mL.

#### 2.9.2. Broth Microdilution Method for Determination of the Minimum Inhibitory Concentration (MIC)

The minimum inhibitory concentrations (MICs) were determined using 96-well microdilution plates, following the methodologies described in CLSI M07 [41]. Briefly, 90 µL of Mueller-Hinton broth was added to each well of sterile, round-bottom plates, followed by 100 µL of the AgSD hydrogels (ρ_hydrogel = 1.29 g/mL or 129 mg) and the commercial cream. Serial dilutions were performed to obtain final concentrations ranging from 1000 to 7.8125 μg/mL for the 1% AgSD hydrogel, and from 1200 to 9.375 μg/mL for the 1.2% AgSD hydrogel. Each well then received 10 µL of a bacterial suspension with a final concentration of 1.5 × 10^8^ CFU/mL. After treatment, the plates were incubated at 35 ± 2 °C for 24 h. The negative control consisted of the bacterial suspension combined with broth only. Antibiotics such as ciprofloxacin hydrochloride (CIPRO), oxacillin (OXA), and ceftazidime (CFT), purchased from Sigma-Aldrich^®^, Merck KGaA, Darmstadt, Germany, were used as positive controls at a concentration of 200 μg/mL. After incubation, 20 µL of 2,3,5-triphenyltetrazolium chloride (TTC, 2% in sterile water) was added, and visual readings were taken after 1 h of incubation. All experiments were performed in triplicate.

#### 2.9.3. Minimum Bactericidal Concentration (MBC)

To determine the minimum bactericidal concentration (MBC), 10 µL from each well showing no visible growth, along with one dilution above and one below the MIC, was subcultured onto Mueller-Hinton Agar (MHA). These subcultures were incubated at 35 ± 2 °C for 24 h. The MBC was defined as the lowest concentration that prevented visible bacterial growth, i.e., the lowest concentration of the antimicrobial agent that resulted in the complete absence of bacterial colonies on the agar plates.

### 2.10. Evaluation of Antibiofilm Activity

The antibiofilm activity assay was conducted according to a methodology adapted from Oliveira et al. (2023) [42], using *S. aureus*, *E. coli*, and *P. aeruginosa* strains. Initially, 24 h bacterial cultures in MHB were standardized to a turbidity equivalent to the 0.5 McFarland standard (1.0 × 10^6^ to 5.0 × 10^6^ CFU/mL), confirmed spectrophotometrically at 600 nm, and then inoculated into 96-well microdilution plates (100 µL/well, flat-bottom, sterile polystyrene plates). Subsequently, 100 µL of the AgSD hydrogel formulations were added to each well at the tested concentration and incubated at 35 ± 2 °C for 24 h to assess their activity against biofilm formation. After incubation, the supernatant was carefully removed, and the wells were washed with sterile saline solution (0.85% NaCl) to eliminate non-adherent cells. The plates were then air-dried at room temperature (25 °C) for 20 min. Each assay included a positive control (MHB + inoculum) and a negative control (MHB only). Biofilm biomass quantification was performed following a modified protocol by Munusamy et al. (2018) [43], involving (a) removal of the treatment medium; (b) triple washing with 0.85% NaCl; (c) drying in an oven at 40 °C for 20 min; and (d) measurement of the residual biomass using crystal violet staining. Biofilms were stained with 0.1% (*w/v*) crystal violet for 15 min, followed by rinsing with distilled water to remove excess dye, and the bound dye was solubilized in 95% ethanol. The results were expressed as the percentage of biofilm formation inhibition relative to the positive control, calculated as % Inhibition = [(OD control − OD treated)/OD control] × 100. This allowed evaluation of the effectiveness of the AgSD hydrogels in preventing bacterial biofilm development.

### 2.11. Statistical Analysis

All experiments were performed in triplicate, and the results were presented as mean ± standard deviation (SD). Data analysis was performed using one-way or two-way analysis of variance (ANOVA) to determine statistical significance, with a *p*-value < 0.05 considered significant. GraphPad Prism (version 10.6, San Diego, CA, USA) was used for statistical analyses. * and ** indicate *p* < 0.01 and *p* < 0.0001, respectively. Drug release kinetics were analyzed by fitting the experimental data to different mathematical models, including zero-order, first-order, Korsmeyer–Peppas, Peppas–Sahlin, and Weibull equations. The fitting parameters (rate constants, release exponent n, and correlation coefficient r^2^) were calculated to determine the best-fitting model for each formulation. The model with the highest r^2^ value was considered the most appropriate to describe the release mechanism.

## 3. Results and Discussion

### 3.1. Evaluation of Laponite^®^ Concentration and Stirring Time on the Apparent Viscosity of the Formed Hydrogel (Drug-Free)

**Figure 1:**
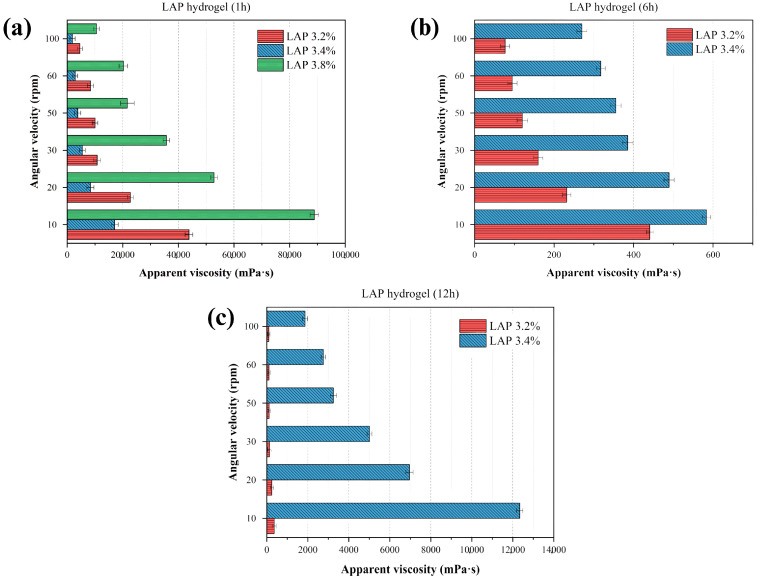
Apparent viscosity of Laponite^®^ (LAP) hydrogels at concentrations of 3.2%, 3.4%, and 3.8% at different time points after preparation: (**a**) 1 h, (**b**) 6 h, and (**c**) 12 h.

Figure 1 presents the quantitative analysis of apparent viscosity, based on the resistance to the rotational movement of a spindle in the fluid. LAP concentrations of 3.2%, 3.4%, and 3.8% were selected according to the range recommended by the raw material supplier and by Brunchi et al. (2024) [32]. As shown in Figure 1a, the apparent viscosity increased significantly with LAP concentration, particularly after 1 h of mixing. This behavior is attributed to the intrinsic structural properties of Laponite^®^, which enable the formation of a repulsive colloidal phase stabilized by long-range electrostatic repulsion and Van der Waals attractive interactions. These characteristics enhance the material’s water absorption capacity, resulting in swelling, volume expansion, and subsequent hydrogel formation, as evidenced by the sharp increase in apparent viscosity (88,870 mPa·s) for the 3.8% LAP formulation, which led to the cessation of analysis due to gel solidification at this concentration and time point.

After 6 h (Figure 1b), the 3.2% and 3.4% formulations exhibited viscosity values much lower than those observed after the first hour, ranging from 440.8 mPa·s to 583.43 mPa·s, even at the lowest shear rates. This behavior may be related to a structural reorganization and partial relaxation of the colloidal network, suggesting that some of the initial interactions are temporary and may be disrupted. However, the system undergoes reorganization over time (Figure 1c), with a subsequent increase in viscosity. In other words, it does not display a linear behavior with respect to solid content over time.

Moreover, it is important to highlight that at concentrations close to the critical gelation limit (between 3.2% and 3.4%), the colloidal network evolves differently over time [32,44]. At 1 h, the 3.4% system exhibits higher viscosity due to the formation of a denser network. However, upon prolonged stirring and aging, this initially rigid structure undergoes partial relaxation and microstructural rupture, leading to a decrease in viscosity at 6 h. In contrast, the 3.2% dispersion, being less dense, maintains a more stable colloidal arrangement, which explains its relatively higher viscosity at 6 h. This inversion of trends reflects the non-linear, time-dependent rheological behavior typical of Laponite^®^ dispersions [18], where structural rearrangements and the rebalancing of electrostatic and Van der Waals forces govern the apparent viscosity profile [44,45].

### 3.2. Macroscopic and Rheological Characterization of LAP@AgSD Hydrogels Prepared by the One-Pot Method

**Figure 2:**
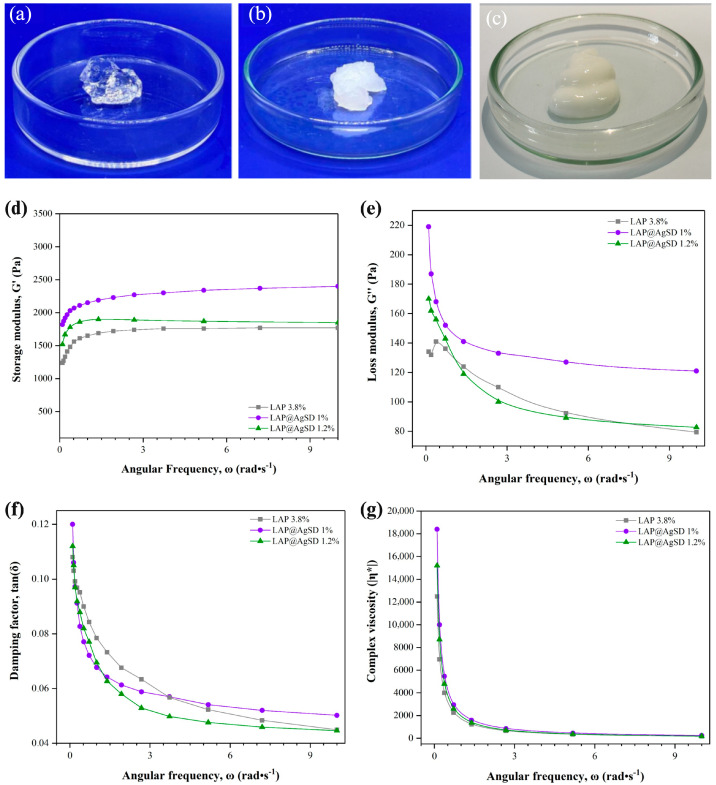
(**a**) LAP 3.8% hydrogel; (**b**) LAP@AgSD hydrogel at 1% (opaque aspect); and (**c**) commercial AgSD cream. Oscillatory rheological characterization of LAP 3.8%, LAP@AgSD 1%, and LAP@AgSD 1.2% hydrogels at 25 ± 0.1 °C using a parallel plate geometry (25 mm diameter, 1 mm gap) in a controlled-stress rheometer (Anton Paar MCR 301): (**d**) storage modulus (G′), representing the elastic component and the ability of the hydrogel to store energy; (**e**) loss modulus (G″), corresponding to the viscous component and energy dissipation during deformation; (**f**) damping factor (tan δ = G″/G′), indicating the balance between viscous and elastic behavior (tan δ < 1, predominantly elastic; tan δ > 1, predominantly viscous); and (**g**) complex viscosity (η*), expressing the overall resistance of the hydrogel to oscillatory deformation.

Figure 2a,b display the macroscopic characteristics of hydrogels formed with drug-free LAP (LAP 3.8%) (Figure 2a) and drug-loaded LAP (LAP@AgSD 1%) (the macroscopic appearance of LAP@AgSD 1.2% is similar to that of LAP@AgSD 1%) (Figure 2b). The LAP 3.8% hydrogel is transparent and clear; however, with the addition of AgSD and subsequent pH adjustment to 5.0, a change in appearance was observed. This is attributed to the presence of electrolytes, particularly those whose ions are strongly hydrated, causing the colloidal material to lose its solvation water to these ions and leading to coagulation, a phenomenon known as “salt-out” precipitation [46]. In addition, Figure 2c shows the appearance of the commercial AgSD cream (CC-AgSD 1%), which presents a dense, homogeneous, and opaque white structure, in contrast to the transparent or semi-opaque aspect observed in the LAP-based hydrogels.

The rheological analysis of LAP hydrogels and those incorporating AgSD is presented in Figure 2c–f. The storage modulus (G′) reflects the elastic behavior of the material, i.e., its ability to store mechanical energy during deformation [47]. It was observed that the LAP 3.8% hydrogel exhibited the lowest G′ values (~1200–1700 Pa), while the AgSD-loaded hydrogels, especially at the 1% concentration, showed higher values (~2000–2500 Pa), indicating greater stiffness and a more pronounced solid-like behavior (Figure 2c). Overall, the incorporation of AgSD increased G′ compared to drug-free LAP, suggesting reinforcement of the three-dimensional network. However, at 1.2%, excessive ionic interactions likely disrupted network homogeneity, slightly reducing G′ relative to the 1% formulation.

The loss modulus (G″), associated with the viscous behavior and energy dissipation [48], was also higher in the AgSD-loaded hydrogels, particularly for the LAP@AgSD 1% formulation, which reached values above 200 Pa at low frequencies (Figure 2d). This behavior suggests the presence of a denser and more interactive internal network, contributing to both the elastic and viscous characteristics of the system. As the frequency increases, G″ decreases for all formulations, which is expected in non-Newtonian viscoelastic systems [49].

The damping factor (tan δ = G″/G′), shown in Figure 2e, expresses the relationship between the viscous and elastic moduli, indicating the degree of viscoelasticity [50]. All hydrogels exhibited tan δ values below 0.12, characterizing a predominantly elastic behavior. The LAP@AgSD 1.2% formulation displayed the lowest tan δ values across the entire frequency range, indicating the stiffest and least dissipative system among those evaluated. In contrast, the LAP 3.8% hydrogel exhibited higher damping factors, especially at low frequencies, suggesting a less cohesive structural network and greater susceptibility to energy dissipation.

The complex viscosity (η*), represented in Figure 2f, also decreased with increasing angular frequency for all formulations, indicating a pseudoplastic behavior typical of structured hydrogels. The LAP@AgSD 1% formulation showed the highest η* values at low frequencies, indicating greater resistance to flow and a more structured system, followed by LAP@AgSD 1.2% and LAP 3.8%. Overall, the rheological data demonstrate that the incorporation of AgSD enhances the stiffness, elasticity, and viscosity of LAP hydrogels, with the LAP@AgSD 1% standing out by exhibiting the most favorable mechanical and structural parameters. These features may support its application as a controlled-release system and topical material with improved mechanical stability.

### 3.3. Thermal Behavior and Drug–Clay Interactions of LAP@AgSD Hydrogels

**Figure 3:**
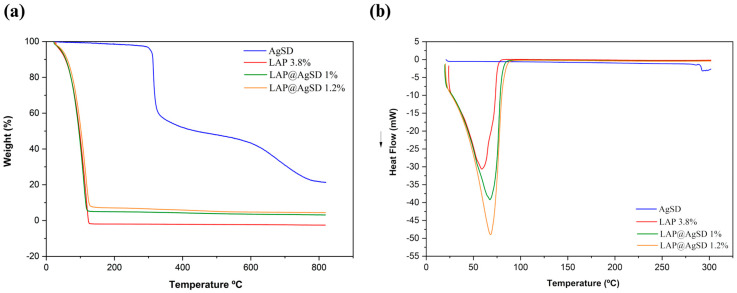
Thermal analysis of AgSD, LAP 3.8%, and AgSD-loaded Laponite^®^ hydrogels (LAP@AgSD 1% and LAP@AgSD 1.2%). (**a**) Thermogravimetric analysis (TGA): weight loss (%) as a function of temperature, showing the degradation profile of pure silver sulfadiazine (AgSD), drug-free hydrogel (LAP 3.8%), and drug-loaded hydrogels; (**b**) Differential scanning calorimetry (DSC): heat flow (mW) versus temperature, highlighting the endothermic and exothermic transitions of the same samples. Abbreviations: AgSD, silver sulfadiazine; LAP, Laponite^®^; LAP@AgSD, Laponite^®^ hydrogel loaded with AgSD.

The thermal behavior of the formulations was evaluated by thermogravimetric analysis (TGA) and differential scanning calorimetry (DSC), as shown in Figure 3a–b. The TGA curves (Figure 3a) demonstrate that the pure drug (AgSD) exhibits two main mass-loss events: the first with an onset temperature (T_onset_) at 275 °C and an endset temperature (T_endset_) at 312.19 °C, associated with the thermal degradation of the aminopyrimidine moiety [51], and the second with a T_onset_ at 319.89 °C and T_endset_ at 419.13 °C, attributed to the degradation of the aromatic ring and the decomposition of sulfur dioxide [52]. These events resulted in a total mass loss exceeding 75%, indicating complete thermal degradation of the drug.

In contrast, the LAP 3.8% hydrogel exhibited a single mass-loss event within the temperature range of 28.72–125.78 °C, corresponding to a Δm% of 99.99%. This behavior is characteristic of clay-based hydrogels composed primarily of physically and structurally bound water, with no significant organic content contributing to degradation at higher temperatures. The hybrid formulations containing AgSD showed distinct thermal profiles. For LAP@AgSD 1%, the first mass-loss stage occurred between 23.60 and 120.74 °C, with a Δm% of 94.24%, whereas for LAP@AgSD 1.2% it ranged from 26.01 °C to 127.70 °C, with a Δm% of 91.36%. The gradual decrease in mass loss with increasing AgSD content confirms the presence of non-volatile drug incorporated into the hydrogel matrix. The shift in onset temperatures and reduced total mass loss indicate partial protection of the drug within the Laponite^®^ network, likely through intermolecular interactions and physical entrapment. These findings suggest that the incorporation of AgSD into the LAP matrix results in a hybrid hydrogel with enhanced thermal stability and increased residual content, providing evidence of successful drug loading.

Complementary information on the thermal behavior of the formulations was obtained through DSC, which showed a shift in the endothermic event to higher temperatures, with peaks observed at 67.34 °C for LAP@AgSD 1% and 68.30 °C for LAP@AgSD 1.2%. This progressive increase suggests stronger water retention within the hydrogel network, likely due to intermolecular interactions between the drug and the clay matrix, such as hydrogen bonding or electrostatic interactions. Notably, the characteristic thermal event of AgSD decomposition around 285 °C [22] was absent in both LAP@AgSD formulations, indicating that the drug is either amorphized or molecularly dispersed within the Laponite^®^ structure. This thermal suppression provides further evidence of successful drug incorporation and strong drug–clay interactions, which can enhance the physical stability and performance of the hydrogel in topical delivery systems.

### 3.4. Molecular Interactions and Surface Charge Modulation in LAP@AgSD Hydrogels

**Figure 4:**
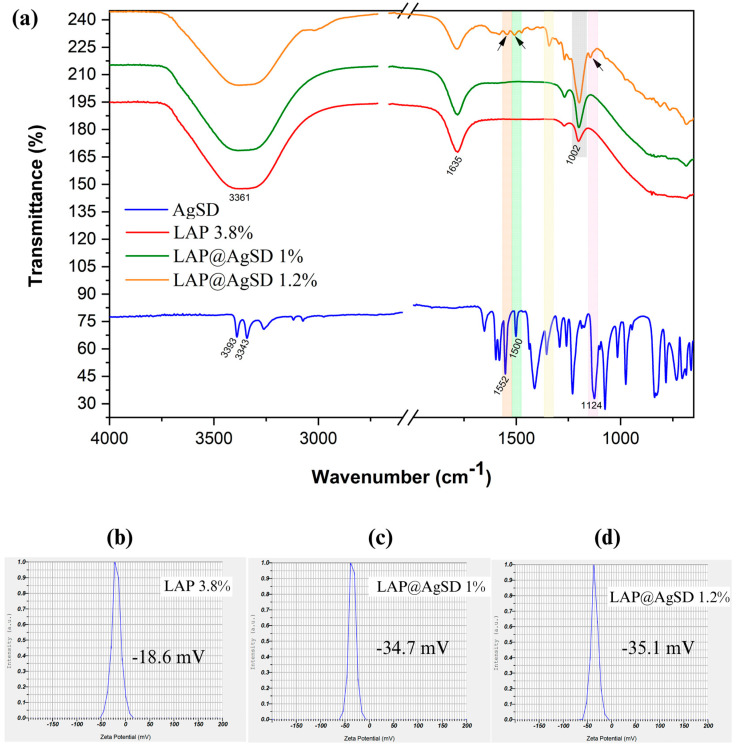
(**a**) Fourier-transform infrared spectroscopy (FTIR) spectra showing characteristic absorption bands of pure silver sulfadiazine (AgSD), drug-free hydrogel (LAP 3.8%), and drug-loaded hydrogels. The arrows indicate the main absorption bands where structural changes associated with LAP–AgSD interactions were observed. Zeta potential (ZP) analysis showing the surface charge distribution of (**b**) LAP 3.8% (−18.6 mV), (**c**) LAP@AgSD 1% (−34.7 mV), and (**d**) LAP@AgSD 1.2% (−35.1 mV). Abbreviations: AgSD, silver sulfadiazine; LAP, Laponite^®^; LAP@AgSD, Laponite^®^ hydrogel loaded with AgSD; FTIR, Fourier-transform infrared spectroscopy; ZP, zeta potential.

Figure 4 shows the FTIR spectra of AgSD, LAP 3.8%, LAP@AgSD 1%, and LAP@AgSD 1.2% (Figure 4a). The spectrum of pure AgSD exhibited characteristic absorption bands at 3343 cm^−1^ and 3393 cm^−1^, corresponding to the stretching vibrations of the primary amine (-NH_2_), along with bands at 1552 cm^−1^ and 1500 cm^−1^, attributed to the C=C stretching of the aromatic pyrimidine ring. A prominent band at 1124 cm^−1^ was assigned to the symmetric stretching of the sulfonyl group (-SO_2_), which is a marker of the structural integrity of the drug [53,54]. The LAP 3.8% spectrum showed typical features of smectite-type clays, including a broad OH stretching band centered at 3361 cm^−1^ related to structural and adsorbed water, an OH bending band at 1635 cm^−1^, a strong Si-O stretching band at 1002 cm^−1^, and a band at 656 cm^−1^ corresponding to Mg-OH-Mg bending [55,56].

In the LAP@AgSD 1.2% formulation, the spectrum revealed the emergence of distinct bands from the drug, including signals in the region of 1500–1550 cm^−1^ and a visible shoulder near 1124 cm^−1^, which were absent or negligible in the lower-concentration formulations. The appearance of these AgSD bands at higher drug concentrations confirms the successful incorporation of the drug into the clay matrix and its structural preservation. The fact that these signals do not shift substantially suggests that the interaction is physical rather than chemical, likely involving hydrogen bonding or electrostatic forces between the drug and the charged surface of Laponite^®^.

The Si-O stretching band at 1002 cm^−1^, assigned to the tetrahedral silicate structure of Laponite^®^, became more intense with increasing AgSD concentration. This enhancement in band intensity may be associated with increased molecular organization or structural ordering of the silicate network upon drug incorporation. According to Kaya et al. (2020) [57], intensification of the Si-O band can reflect changes in the local symmetry and vibrational coupling within the silicate layers due to intercalation or surface adsorption processes. In this case, it suggests that AgSD contributes to a more compact or ordered clay–drug hydrid structure. These spectral findings confirm the presence of AgSD within the Laponite^®^ matrix and support the hypothesis that the drug is physically entrapped without chemical degradation, maintaining its functional groups while establishing stabilizing interactions with the host hydrogel network.

This interpretation is further corroborated by zeta potential measurements (Figure 4b–d), which reflect changes in the surface charge of the hydrogels upon drug incorporation. The LAP 3.8% formulation exhibited a zeta potential of −18.6mV (Figure 4b), indicative of moderate colloidal stability. However, after AgSD incorporation, a marked increase in the negative surface charge was observed, with zeta potential values of −34.7 mV (Figure 4c) and −35.1 mV (Figure 4d) for LAP@AgSD 1% and 1.2%, respectively.

This shift toward more negative values suggests that the sulfonamide and sulfonic acid groups of AgSD contributed additional anionic character to the surface of the hybrid particles, likely through electrostatic interactions with the Laponite^®^ platelets [58]. The enhanced surface charge promotes stronger interparticle repulsion, minimizing aggregation and favoring colloidal stabilization. Moreover, the similarity between the zeta potential values of the two drug-loaded formulations indicates that surface saturation may have been achieved at 1% drug loading, reinforcing the idea of efficient surface coverage and interaction.

### 3.5. Morphological Evaluation of LAP and LAP@AgSD Hydrogels by TEM

**Figure 5:**
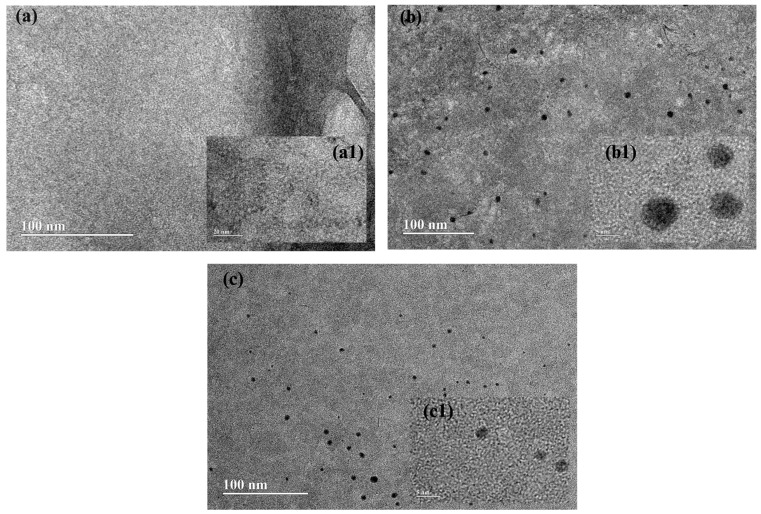
Transmission Electron Microscopy (TEM) of Laponite^®^ hydrogels: (**a**) blank LAP hydrogel (3.8% *w/v*), (**a1**) higher magnification, (**b**) hydrogel loaded with 1% silver sulfadiazine (LAP@AgSD 1%), (**b1**) high-resolution image, (**c**) hydrogel loaded with 1.2% silver sulfadiazine (LAP@AgSD 1.2%), (**c1**) high-resolution image. Scale bars: 100 nm (**a**–**c**), 20 nm (**a1**), 5 nm (**b1**,**c1**).

The morphology of the hydrogels was characterized by Transmission Electron Microscopy (TEM), as presented in Figure 5. The micrographs of the blank LAP 3.8% hydrogel revealed a dense colloidal network, with homogeneous contrast resulting from the superposition of lamellae and small tactoids (Figure 5a). The percolating “house-of-cards” structure (Figure 5(a1)), with high tortuosity and absence of coarse agglomerates, indicates efficient dispersion and matrix stability. For the hydrogel loaded with 1% AgSD (LAP@AgSD 1%) (Figure 5b), the micrographs displayed a homogeneous nanogranular background, typical of overlapped lamellae/tactoids forming a percolating network, with connected lamellar domains and more frequent clear voids, suggesting a less compact mesh. In addition, sparse, nearly spherical dark spots were observed (Figure 5(b1)), distributed throughout the matrix, consistent with silver-rich domains (concentrated AgSD and/or Ag nanoparticles generated by reduction during preparation/beam), with no evidence of large precipitates or dense domains >50–100 nm, indicating good drug dispersion within the gel. Conversely, the hydrogel loaded with 1.2% AgSD (LAP@AgSD 1.2%) (Figure 5c) exhibited more extensive dense domains, suggesting a more compact mesh, i.e., higher tortuosity, with the same scattered electron-dense spots (Ag-rich regions) (Figure 5(c1)). The particle density was visibly higher than in the LAP@AgSD 1% sample, consistent with ζ ≈ −35.1 mV (good colloidal stability and low aggregation tendency). No coarse agglomerates were observed. In both cases, the morphology confirmed the presence of a solid silver reservoir immobilized within a dense colloidal network. This morphological feature may be attributed to stronger chemical interactions between AgSD and the Laponite^®^ lamellae, involving electrostatic forces, hydrogen bonding, and possible coordination of Ag^+^ ions with oxygenated sites on the clay surface. Such interactions likely contribute to the formation of a denser and more organized colloidal network, enhancing matrix stability.

### 3.6. In Vitro Drug Release Profile Using Franz Diffusion Cells

**Figure 6:**
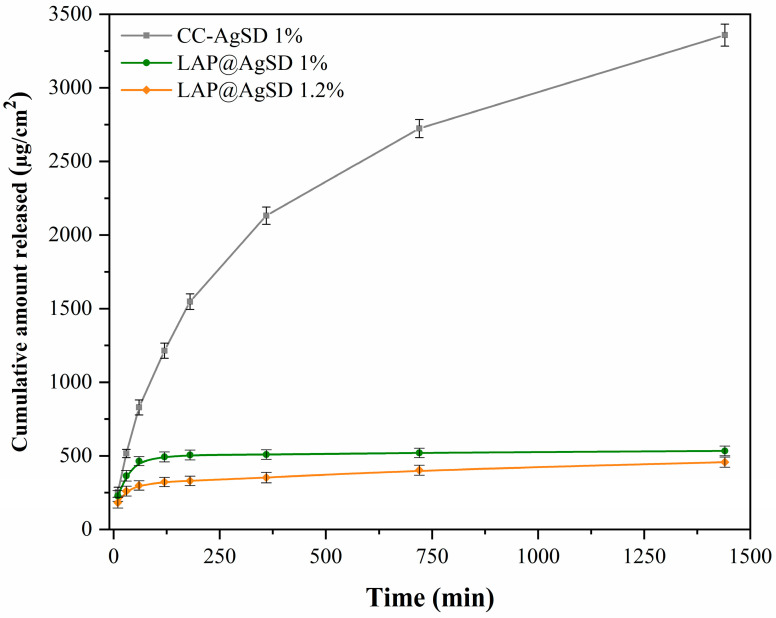
Cumulative amount of AgSD released (µg/cm^2^) from the commercial cream (CC-AgSD 1%) and from Laponite^®^ hydrogels loaded with AgSD (LAP@AgSD 1% and LAP@AgSD 1.2%), over 24 h. Results are expressed as mean ± SD (n = 6).

The in vitro release profile comparing the commercial silver sulfadiazine cream (CC-AgSD 1%) and the LAP@AgSD 1% and LAP@AgSD 1.2% hydrogels is shown in Figure 6. The commercial cream exhibited a rapid release, reaching 829.00 µg/cm^2^ (23.13%) within just 60 min and 3358.13 µg/cm^2^ (35.30%) after 24 h, which is typical of conventional systems lacking structural diffusion barriers [59]. In contrast, the LAP@AgSD 1% and 1.2% hydrogels released significantly lower amounts of drug over the same period, with 5.26% and 4.21% at 60 min, respectively, and 5.61% and 4.81% after 24 h. This behavior characterizes a controlled-release system, in which the LAP colloidal matrix acts as a physical and interactive barrier, restricting drug release [60]. It is noteworthy that the hydrogel containing a higher concentration of AgSD, LAP@AgSD 1.2%, released less drug than LAP@AgSD 1%. This may be attributed to the higher negative charge density and physicochemical interactions between the drug and the LAP network [61], as suggested by the zeta potential results. The increased negative surface charge may promote greater adsorption or retention of the drug at LAP binding sites, thereby hindering its diffusion into the external medium. These morphological observations help explain the release profiles, since the denser and more tortuous network of LAP@AgSD 1.2% hinders drug diffusion, leading to slower and more sustained release, whereas the less compact LAP@AgSD 1% matrix allows a slightly faster but still controlled release compared to the commercial cream. 

Table 1 presents the fitting parameters of the mathematical models applied to the in vitro release data of silver sulfadiazine (AgSD) from the LAP@AgSD 1% and 1.2% hydrogels, enabling the understanding of the predominant release mechanisms in these systems compared to the commercial cream. The mathematical models incorporate one or more parameters in their equations that may or may not be related to physicochemical properties, aiming to describe the phenomena involved in drug release.

Among the models evaluated, the Weibull model showed the best fit for both formulations, with correlation coefficients (r^2^) of 0.9979 for LAP@AgSD 1% and 0.9981 for LAP@AgSD 1.2%. This model is frequently associated with diffusion-controlled systems and structural diffusion barriers [33]. The β value < 1 (0.70 and 0.67) suggests a release profile with a decreasing rate over time, which is typical of diffusion-controlled mechanisms in rigid or hydrated matrices, such as Laponite^®^-based hydrogels [34,62]. 

Furthermore, when compared to the commercial cream, which followed a first-order release profile, the LAP@AgSD hydrogels demonstrated a markedly different behavior. The Weibull model fitting with β < 1 indicates a sustained release governed by diffusion through the hydrated Laponite^®^ matrix, in contrast to the rapid release observed for the cream. Clinically, such sustained release is highly desirable, as it can prolong the therapeutic window, reduce the frequency of dressing changes, and minimize systemic absorption and potential side effects of AgSD. This controlled-release behavior highlights the potential of LAP-based hydrogels as advanced topical delivery systems for the management of burns and chronic wounds, where maintenance of antimicrobial activity over extended periods is critical [61,63,64].

It is worth noting that the Peppas–Sahlin model also exhibited good fits (r^2^ > 0.99) and revealed that the exponent m ≈ 0.45–0.46 falls within the characteristic range of Fickian diffusion, meaning that drug transport occurs predominantly through diffusion of the active agent across the hydrated matrix. The k_1_ parameter, related to diffusion, was considerably higher than k_2_, associated with matrix relaxation, which presented values close to zero or negative. This indicates that AgSD release is mainly governed by diffusion, with minimal contribution from relaxation processes within the structural network of Laponite^®^.

### 3.7. Assessment of Antibacterial Efficacy of Silver Sulfadiazine-Loaded Laponite^®^ Hydrogels

Table 2 presents the antimicrobial potential of the developed hydrogels, which was evaluated by determining the minimum inhibitory concentration (MIC) against three representative bacterial strains: *Escherichia coli* (Gram-negative), *Pseudomonas aeruginosa* (Gram-negative), and *Staphylococcus aureus* (Gram-positive). Hydrogels formulated with Laponite^®^ (LAP) alone did not exhibit antibacterial activity against any of the tested strains, corroborating previous findings in the literature [22].

In contrast, the hydrogels incorporating AgSD at concentrations of 1% and 1.2% demonstrated effective antibacterial activity, with a dose-dependent improvement. For the LAP@AgSD 1% formulation, MIC values were 62.5 μg/mL for *E. coli*, 31.25 μg/mL for *P. aeruginosa*, and 18.75 μg/mL for *S. aureus*. When the AgSD concentration was increased to 1.2%, the MIC decreased to 37.5 μg/mL for *E. coli* and to 18.75 μg/mL for *P. aeruginosa*, while remaining unchanged for *S. aureus* (18.75 μg/mL), indicating enhanced antibacterial efficacy.

This increase in antimicrobial activity is attributed to the higher availability of silver ions (Ag^+^), which disrupt bacterial membranes, denature essential enzymes through interaction with thiol groups, induce oxidative stress, and interfere with DNA replication [65,66]. Additionally, the presence of LAP contributes to the prolonged and controlled release of AgSD, enhancing local drug retention at the infection site. These findings are consistent with the TEM observations, in which the homogeneous dispersion of silver-rich domains and the absence of large aggregates in the LAP@AgSD hydrogels ensure a gradual and sustained release of Ag^+^, thereby supporting the prolonged antimicrobial activity observed. The observed improvement in efficacy with increasing AgSD concentration suggests that the LAP-based hydrogel matrix is an efficient platform for the sustained topical delivery of silver sulfadiazine, particularly for the treatment of contaminated wounds.

### 3.8. Inhibition of Bacterial Biofilms by Silver Sulfadiazine-Loaded Laponite^®^ Hydrogels

**Figure 7:**
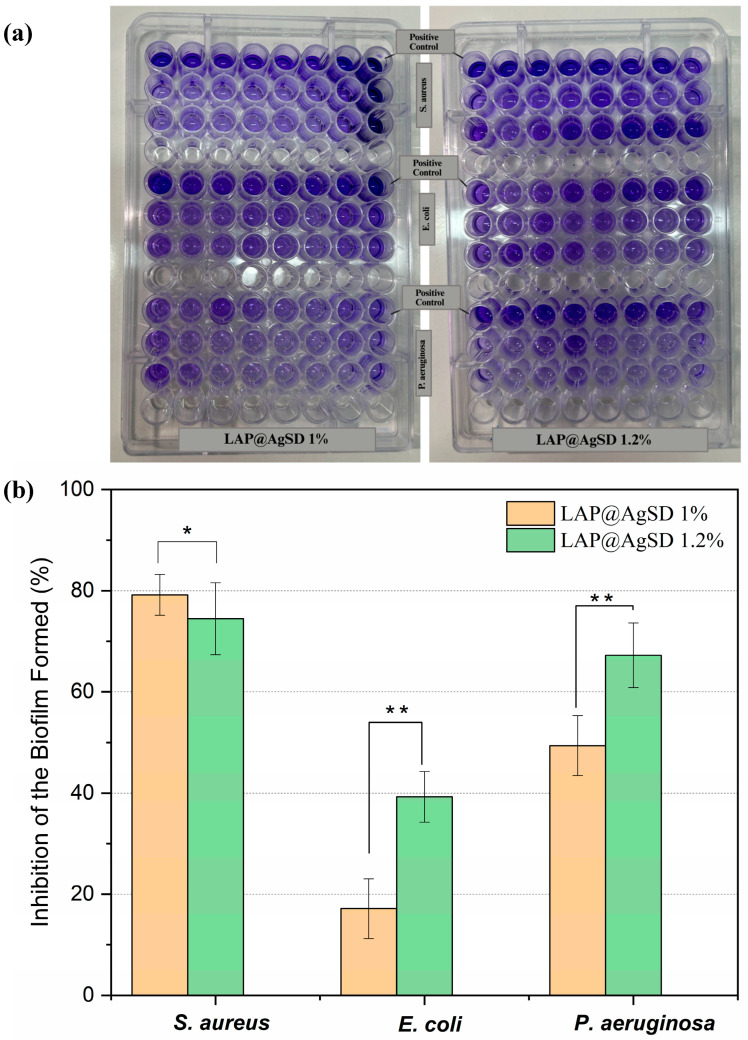
(**a**) Representative images of biofilm formation after treatment with LAP@AgSD 1% and LAP@AgSD 1.2% against *Staphylococcus aureus*, *Escherichia coli*, and *Pseudomonas aeruginosa*, compared with the positive controls. (**b**) Percentage of biofilm inhibition by LAP@AgSD 1% and LAP@AgSD 1.2% against bacterial strains of clinical interest. Results are displayed as mean ± standard deviation (SD), n = 3. * = *p* < 0.01, ** = *p* < 0.0001.

A biofilm is a 3D aggregate of microorganisms adhered to a surface and surrounded by extracellular polymeric substances (EPSs) consisting of proteins, polysaccharides, lipids, and deoxyribonucleic acids (DNA) [67]. Accordingly, the antibiofilm efficacy against biofilms formed by *Staphylococcus aureus*, *Pseudomonas aeruginosa*, and *Escherichia coli* strains was evaluated using hydrogels containing AgSD (LAP@AgSD 1% and 1.2%) over a 24 h period (Figure 7a). For biofilms formed by *S. aureus*, both hydrogels exhibited approximately 79.18% inhibition of the biomass (Figure 7b). This result suggests that, for this Gram-positive strain, the 1% concentration is already sufficient to exert an effective antibiofilm action, possibly due to the efficient penetration of Ag^+^ ions into the biofilm and the high affinity of AgSD for extracellular matrix components of *S. aureus*. In contrast, for *E. coli* and *P. aeruginosa*, a dose-dependent pattern was observed, with LAP@AgSD 1% showing lower activity (17.16% and 43.37%, respectively), while LAP@AgSD 1.2% showed nearly double the inhibition (39.93% and 67.22%). This may be related to the presence of the lipopolysaccharide-rich outer membrane in *E. coli* and *P. aeruginosa*, which can hinder silver penetration at lower concentrations [68]. Additionally, *P. aeruginosa* can form thick biofilms rich in exopolysaccharides, and the enhanced performance of the more concentrated formulation may be associated with the greater availability of silver over time [69].

However, this dose–effect relationship is not necessarily more effective for all types of biofilms, which is relevant when considering toxicity and cost. It is also important to highlight that the sustained release provided by the LAP system may be crucial for maintaining long-term antibiofilm effects, especially in contexts such as the treatment of chronic wounds, where frequent reapplication of conventional medications is limited.

### 3.9. Limitations of the Study

This study has some limitations that should be acknowledged. First, only in vitro assays were performed, and in vivo wound-healing and toxicity data are still lacking. Further studies in animal models are necessary to confirm the therapeutic efficacy and safety profile of LAP@AgSD hydrogels. Second, no long-term stability studies under accelerated or real-time conditions were conducted, which limits conclusions about the shelf-life and storage requirements of the formulations. Finally, cytotoxicity evaluation using established mammalian cell lines, such as fibroblasts or keratinocytes, was not performed, which restricts broader extrapolation of the biocompatibility findings. These aspects should be addressed in future investigations to strengthen the translational potential of the proposed system.

## 4. Conclusions

The results of this study demonstrate that the use of Laponite^®^ as a single excipient enables the development of a practical, fast, and reproducible hydrogel system for the topical administration of silver sulfadiazine (AgSD), eliminating the need for complex excipient combinations. It is important to highlight that the incorporation of AgSD significantly improved the elastic modulus (G′) and structural integrity, ensuring mechanical robustness for topical application while maintaining spreadability and stability. This mechanism, combined with a diffusion-controlled release behavior, especially in the LAP@AgSD 1% formulation, provides effective availability, bactericidal action against both Gram-positive and Gram-negative strains, and a significant reduction in biofilms. Therefore, these results position LAP@AgSD 1% as a clinically relevant and scalable formulation for the treatment of infected or chronic wounds, particularly those involving biofilm-forming pathogens. The simplicity of its composition and manufacturing process reinforces its potential to be translated into cost-effective wound treatment products. Future studies should include in vivo validation to confirm the therapeutic efficacy and safety profile, as well as further investigations aimed at clinical translation and large-scale application.

## Figures and Tables

**Table 1:** Kinetic parameters of AgSD release from commercial cream (CC-AgSD 1%) and Laponite^®^-based hydrogels (LAP@AgSD 1% and LAP@AgSD 1.2%). Abbreviations: AgSD, silver sulfadiazine; LAP, Laponite^®^; LAP@AgSD, Laponite^®^ hydrogel loaded with AgSD; CC-AgSD, commercial cream containing 1% AgSD; k_0_, zero-order release constant; k_1_, first-order release constant; K_kp_- Korsmeyer–Peppas release constant; n, release exponent; k_1_ and k_2_, Peppas–Sahlin constants for Fickian diffusion and polymer relaxation, respectively; α, scale parameter of the Weibull model; β, shape parameter of the Weibull model; r^2^, coefficient of determination.

		Formulations		
Models	Parameters	LAP@AgSD 1%	LAP@AgSD 1.2%	CC-AgSD 1%
**Zero-order**	**k_0_ (** **μ** **g.min^−1^)**	10.57	7.61	42.24
	**r^2^**	0.8679	0.8905	0.9533
**First-order**	**k_1_ (min^−1^) × 10^−3^**	0.0019	0.0019	0.0020
	**r^2^**	0.9714	0.9792	**0.9968**
**Korsmeyer–Peppas**	**K_kp_(min^−n^) × 10^−3^**	272,72	214.58	163.25
	***n***	0.62	0.60	0.87
	**r^2^**	0.9383	0.9570	0.9676
**Peppas–Sahlin**	**k_1_(min** **^−^** **^m^** **) × 10^−3^**	1047.35	705.62	1637.43
	**k_2_(min^−2m^) × 10^−3^**	−13.23	−7.78	31.55
	**r^2^**	**0.9943**	**0.9965**	**0.9928**
**Weibull**	**α (min)**	55.39	49.70	479.84
	**β**	0.70	0.67	0.99
	**r^2^**	**0.9979**	**0.9981**	**0.9923**
**Best Fit**		**Weibull**	**Weibull**	**First-order**

**Table 2:** Minimum inhibitory concentration (MIC) values of Laponite^®^-based hydrogels containing silver sulfadiazine (LAP@AgSD 1% and 1.2%) against *Escherichia coli*, *Pseudomonas aeruginosa*, and *Staphylococcus aureus*.

Microorganisms	MIC/MBC (μg/mL)			
	LAP@AgSD 1%	LAP@AgSD 1.2%	CC-AgSD l%	LAP
*Escherichia coli* (ATCC 25922)	**62.5/62.5**	**37.5/18.75**	**7.81/7.81**	**na**
*Pseudomonas aeruginosa* *(ATCC 27853)*	**31.25/31.25**	**18.75/18.75**	**7.81/7.81**	**na**
*Staphylococcus aureus* *(ATCC 25923)*	**18.75/31.25**	**18.75/37.5**	**7.81/7.81**	**na**

ATCC, American Type Culture Collection; MIC, minimum inhibitory concentration; MBC, minimum bactericidal concentration; AgSD, silver sulfadiazine; LAP, Laponite^®^; na, no activity.

## Data Availability

The data supporting the findings of this study are available within the article and its ESI. Requests for access to additional data should be directed to the corresponding author.

## References

[1] Yakupu A., Aimaier R., Yuan B., Chen B., Cheng J., Zhao Y., Peng Y., Dong J., Lu S. (2023). The Burden of Skin and Subcutaneous Diseases: Findings from the Global Burden of Disease Study 2019. Front. Public Health.

[2] Cavallo I., Sivori F., Mastrofrancesco A., Abril E., Pontone M., Di Domenico E.G., Pimpinelli F. (2024). Bacterial Biofilm in Chronic Wounds and Possible Therapeutic Approaches. Biology.

[3] Delir S., Sirousazar M., Kheiri F. (2020). Clindamycin Releasing Bionanocomposite Hydrogels as Potential Wound Dressings for the Treatment of Infected Wounds. J. Biomater. Sci. Polym. Ed..

[4] Lowe A.S., Walker M.D., Cowan R., Baxter G.D. (2001). Therapeutic Ultrasound and Wound Closure: Lack of Healing Effect on x-Ray Irradiated Wounds in Murine Skin. Arch. Phys. Med. Rehabil..

[5] WHO (2023). World Health Organization: Geneva, Switzerland, 2023.List. The Selection and Use of Essential Medicines 2023: Executive Summary of the Report of the 24th WHO Expert Committee on the Selection and Use of Essential Medicines.

[6] Federman D.G., Dardik A., Shapshak D., Ueno C.M., Masterson L., Hopf H.W., Abdullah N., Junkins S., Mostow E.N. (2024). Wound Healing Society 2023 Update on Guidelines for Arterial Ulcers. Wound Repair Regen..

[7] Wounds UK (2017). Best Practice Statement Making Day-to-Day Management of Biofilm Simple.

[8] de Melo D.F., Guedes G.G., de Carvalho Moreira L.M.C., Oshiro-Júnior J.A., de Lima Damasceno B.P.G. (2023). Physicochemical Characterization of Silver Sulfadiazine in Polymeric Wound Dressings. Curr. Pharm. Des..

[9] Abo El-Enin H.A., Ali I.H., Naguib I.A., Tolba N.S., Abdel-Bar H.M. (2025). Augmented Silver Sulfadiazine Nanostructured Lipid Carriers Impregnated Collagen Sponge for Promoting Burn Wound Healing. Int. J. Biol. Macromol..

[10] Fatima Q.U.A., Ahmed N., Siddiqui B., Rehman A., ul Haq I., Khan G.M., Elaissari A. (2022). Enhanced Antimicrobial Activity of Silver Sulfadiazine Cosmetotherapeutic Nanolotion for Burn Infections. Cosmetics.

[11] Munhoz D.R., Bernardo M.P., Malafatti J.O.D., Moreira F.K.V., Mattoso L.H.C. (2019). Alginate Films Functionalized with Silver Sulfadiazine-Loaded [Mg-Al] Layered Double Hydroxide as Antimicrobial Wound Dressing. Int. J. Biol. Macromol..

[12] He S., Liu J., He S., Liu A., Shao W. (2022). Double Crosslinked Polyvinyl Alcohol/Gelatin/Silver Sulfadiazine Sponges with Excellent Antibacterial Performance. Colloids Surf. A Physicochem. Eng. Asp..

[13] Mutlu-Ağardan N.B., Tort S., Aydoğduoğlu Ş., Kıymacı M.E. (2022). A New Insight to Silver Sulfadiazine Antibacterial Dressings: Nanoparticle-Loaded Nanofibers for Controlled Drug Delivery. AAPS PharmSciTech.

[14] Zölß C., Cech J.D. (2016). Efficacy of a New Multifunctional Surfactant-Based Biomaterial Dressing with 1% Silver Sulphadiazine in Chronic Wounds. Int. Wound J..

[15] Soltani S., Akhbari K., Phuruangrat A. (2022). Improved Antibacterial Activity by Incorporation of Silver Sulfadiazine on Nanoporous Cu-BTC Metal-Organic-Framework. Inorganica Chim. Acta.

[16] Samoylenko O., Korotych O., Manilo M., Samchenko Y., Shlyakhovenko V., Lebovka N. (2022). Biomedical Applications of Laponite^®^-Based Nanomaterials and Formulations. Springer Proc. Phys..

[17] Tomás H., Alves C.S., Rodrigues J. (2017). Laponite^®^: A Key Nanoplatform for Biomedical Applications?. Nanomedicine.

[18] Suterio N., Bazzo G.C., Rauber G.S., Silva A.H., Caon T., Parize A.L., Creczynski-Pasa T.B., Stulzer H.K. (2022). Laponite^®^ Gel Formulation Containing Simvastatin for Melanoma Treatment. Appl. Clay Sci..

[19] Munoz-Perez E., Perez-Valle A., Igartua M., Santos-Vizcaino E., Hernandez R.M. (2023). High Resolution and Fidelity 3D Printing of Laponite and Alginate Ink Hydrogels for Tunable Biomedical Applications. Biomater. Adv..

[20] Dávila J.L., d’Ávila M.A. (2017). Laponite as a Rheology Modifier of Alginate Solutions: Physical Gelation and Aging Evolution. Carbohydr. Polym..

[21] Bazmi Zeynabad F., Abbaszad Rafi A., Mahkam M., Taheri H. (2016). Preparation of New PH-Sensitive Nanocomposites through in Situ Copolymerization of Methacrylic Acid with Ionic Liquid-Modified Laponite Clay. Polym. Plast. Technol. Eng..

[22] Ghadiri M., Chrzanowski W., Rohanizadeh R. (2014). Antibiotic Eluting Clay Mineral (Laponite^®^) for Wound Healing Application: An in Vitro Study. J. Mater. Sci. Mater. Med..

[23] Pacelli S., Paolicelli P., Moretti G., Petralito S., Di Giacomo S., Vitalone A., Casadei M.A. (2016). Gellan Gum Methacrylate and Laponite as an Innovative Nanocomposite Hydrogel for Biomedical Applications. Eur. Polym. J..

[24] Zhou R., Zhang W., Zhang Y., Wu X., Huang J., Bo R., Liu M., Yu J., Li J. (2024). Laponite/Lactoferrin Hydrogel Loaded with Eugenol for Methicillin-Resistant Staphylococcus Aureus-Infected Chronic Skin Wound Healing. J. Tissue Viability.

[25] Chen L., Li G., Chen Y., Zeng H., Mao Z., Liu L., Wang X., Xu S. (2022). Thixotropy research of laponite-hydrogel composites for water shutoff in horizontal wells. J. Pet. Sci. Eng..

[26] Long L., Ma X., Zhang H., Lan C. (2025). Novel Preparation of Laponite Based Theranostic Silver Nanocomposite for Drug Delivery, Radical Scavenging and Healing Efficiency for Wound Care Management after Surgery. Regen. Ther..

[27] Hu Y., Xu W., Sun L., Ma X., Zhou P., Zhang C., Cai R., Wang X., Yang H., Tao G. (2025). Multifunctional Injectable Hydrogel Incorporating EGCG-Cu Complexes for Synergistic Antibacterial, Immunomodulatory, and Osteogenic Therapy in Periodontitis. Mater. Today Bio.

[28] Micó-Vicent B., Romero E.P., Bermejo R., Jordán-Núñez J., Viqueira V., Pérez J. (2021). Using Laminar Nanoclays for Phycocyanin and Phycoerythrin Stabilization as New Natural Hybrid Pigments from Microalgae Extraction. Appl. Sci..

[29] Qu B., Xue J., Luo Y. (2021). Self-Assembled Caseinate-Laponite^®^ Nanocomposites for Curcumin Delivery. Food Chem..

[30] Ferreira C.M., da Silva G.J. (2023). Absorption of Essential Oils in Laponite: Stability Enhancement and Structural Characteristics. Appl. Clay Sci..

[31] Imbriano A., Mendico M., Primavilla S., Carafa M., Perioli L., Ricci M., Di Michele A., Viseras C., Pagano C., Villén F.G. (2025). Development of Nanoemulgel System Based on Opuntia Ficus-Indica (L.) Seed Oil and Nanoclays: Formulation, Characterization and Application for Wound Treatment. J. Drug Deliv. Sci. Technol..

[32] Brunchi C.-E., Morariu S. (2024). Laponite^®^—From Dispersion to Gel—Structure, Properties, and Applications. Molecules.

[33] de Jesus Martin-Camacho U., Rodríguez-Barajas N., Sánchez-Burgos J.A., Pérez-Larios A. (2023). Weibull β Value for the Discernment of Drug Release Mechanism of PLGA Particles. Int. J. Pharm..

[34] Siepmann J., Siepmann F. (2012). Modeling of Diffusion Controlled Drug Delivery. J. Control. Release.

[35] Moreira V.M., Leite J.M.D.S., Medeiros K.D.A., de Assis K.M.A., Borges J.C., Santana L.M.B., Moreira L.M.C.d.C., Alves L.P., de Oliveira T.K.B., de Souza da Silveira J.W. (2023). Pentoxifylline/Chitosan Films on Wound Healing: In Vitro/In Vivo Evaluation. Pharmaceutics.

[36] Ritger P.L., Peppas N.A. (1987). A Simple Equation for Description of Solute Release I. Fickian and Non-Fickian Release from Non-Swellable Devices in the Form of Slabs, Spheres, Cylinders or Discs. J. Control. Release.

[37] Noves A., Whitney W.R. (1897). The Rate of Solution of Solid Substances in Their Onw Solutions. J. Am. Chem. Soc..

[38] Korsmeyer R.W., Gurny R., Doelker E., Buri P., Peppas N.A. (1983). Mechanisms of Solute Release from Porous Hydrophilic Polymers. Int. J. Pharm..

[39] Peppas N.A., Sahlin J.J. (1989). A Simple Equation for the Description of Solute Release. III. Coupling of Diffusion and Relaxation. Int. J. Pharm..

[40] Bloor J.R., Morrison J.C. (1972). Effects of Solubilization on Drug Diffusion. J. Pharm. Pharmacol..

[41] Clinical and Laboratory Standards (CLSI) (2018). Methods for Dilution Antimicrobial Susceptibility Tests for Bacteria That Grow Aerobically.

[42] De Oliveira G.D., da Rocha W.R.V., Rodrigues J.F.B., Alves H.D.S. (2023). Synergistic and Antibiofilm Effects of the Essential Oil from Croton Conduplicatus (Euphorbiaceae) against Methicillin-Resistant Staphylococcus Aureus. Pharmaceuticals.

[43] Munusamy K., Vadivelu J., Tay S.T. (2018). A Study on Candida Biofilm Growth Characteristics and Its Susceptibility to Aureobasidin A. Rev. Iberoam. Micol..

[44] Ruzicka B., Zaccarelli E. (2011). A Fresh Look at the Laponite Phase Diagram. Soft Matter.

[45] Labanda J., Llorens J. (2008). Effect of Aging Time on the Rheology of Laponite Dispersions. Colloids Surf. A Physicochem. Eng. Asp..

[46] Bujok S., Konefał M., Konefał R., Nevoralová M., Bednarz S., Mielczarek K., Beneš H. (2022). Insight into the Aqueous Laponite^®^ Nanodispersions for Self-Assembled Poly(Itaconic Acid) Nanocomposite Hydrogels: The Effect of Multivalent Phosphate Dispersants. J. Colloid. Interface Sci..

[47] Ramli H., Zainal N.F.A., Hess M., Chan C.H. (2022). Basic Principle and Good Practices of Rheology for Polymers for Teachers and Beginners. Chem. Teach. Int..

[48] Stojkov G., Niyazov Z., Picchioni F., Bose R.K. (2021). Relationship between Structure and Rheology of Hydrogels for Various Applications. Gels.

[49] Calienni M.N., Martínez L.M., Izquierdo M.C., Alonso S.D.V., Montanari J. (2023). Rheological and Viscoelastic Analysis of Hybrid Formulations for Topical Application. Pharmaceutics.

[50] Vincová A., Šantová K., Kůrová V., Kratochvílová A., Halámková V., Suchánková M., Lorencová E., Sumczynski D., Salek R.N. (2023). The Impact of Divergent Algal Hydrocolloids Addition on the Physicochemical, Viscoelastic, Textural, and Organoleptic Properties of Cream Cheese Products. Foods.

[51] Águila-Rosas J., Quirino-Barreda T., Leyva-Gómez G., González-Zamora E., Ibarra I.A., Lima E. (2020). Sulfadiazine Hosted in MIL-53(Al) as a Biocide Topical Delivery System. RSC Adv..

[52] Yassue-Cordeiro P.H., Zandonai C.H., Genesi B.P., Lopes P.S., Sanchez-Lopez E., Garcia M.L., Fernandes-Machado N.R.C., Severino P., Souto E.B., da Silva C.F. (2019). Development of Chitosan/Silver Sulfadiazine/Zeolite Composite Films for Wound Dressing. Pharmaceutics.

[53] Singh R., Roopmani P., Chauhan M., Basu S.M., Deeksha W., Kazem M.D., Hazra S., Rajakumara E., Giri J. (2022). Silver Sulfadiazine Loaded Core-Shell Airbrushed Nanofibers for Burn Wound Healing Application. Int. J. Pharm..

[54] Chouhan D., Mandal B.B. (2020). Silk Biomaterials in Wound Healing and Skin Regeneration Therapeutics: From Bench to Bedside. Acta Biomater..

[55] Magalhães L.S.S.M., Andrade D.B., Bezerra R.D.S., Morais A.I.S., Oliveira F.C., Rizzo M.S., Silva-Filho E.C., Lobo A.O. (2022). Nanocomposite Hydrogel Produced from PEGDA and Laponite for Bone Regeneration. J. Funct. Biomater..

[56] Meena M., Yadav I., Barani P.K., Gajjar D., Joshi A., Seshadri S., Dhanka M. (2025). Zingerone Nanoparticle and Laponite–Embedded Natural Gum Based Injectable Hydrogel for Fast Track Wound Repair. Colloids Surf. A Physicochem. Eng. Asp..

[57] Kaya H., Ngo D., Gin S., Kim S.H. (2020). Spectral Changes in Si–O–Si Stretching Band of Porous Glass Network upon Ingress of Water. J. Non-Cryst. Solids.

[58] Bormio Nunes J.H., Hideki Nakahata D., Corbi P.P., Ferraz de Paiva R.E. (2023). Beyond Silver Sulfadiazine: A Dive into More than 50 Years of Research and Development on Metal Complexes of Sulfonamides in Medicinal Inorganic Chemistry. Coord. Chem. Rev..

[59] Adepu S., Ramakrishna S. (2021). Controlled Drug Delivery Systems: Current Status and Future Directions. Molecules.

[60] Ojha S., Sharma S., Mishra S. (2023). Hydrogels as Potential Controlled Drug Delivery System: Drug Release Mechanism and Applications. Nanosci. Nanotechnol. Asia.

[61] Kiaee G., Dimitrakakis N., Sharifzadeh S., Kim H.J., Avery R.K., Moghaddam K.M., Haghniaz R., Yalcintas E.P., de Barros N.R., Karamikamkar S. (2022). Laponite-Based Nanomaterials for Drug Delivery. Adv. Healthc. Mater..

[62] Casault S., Slater G.W. (2008). Systematic Characterization of Drug Release Profiles from Finite-Sized Hydrogels. Phys. A Stat. Mech. Its Appl..

[63] Stealey S.T., Gaharwar A.K., Zustiak S.P. (2023). Laponite-Based Nanocomposite Hydrogels for Drug Delivery Applications. Pharmaceuticals.

[64] Alberts A., Moldoveanu E.T., Niculescu A.G., Grumezescu A.M. (2025). Hydrogels for Wound Dressings: Applications in Burn Treatment and Chronic Wound Care. J. Compos. Sci..

[65] Khina A.G., Krutyakov Y.A. (2021). Similarities and Differences in the Mechanism of Antibacterial Action of Silver Ions and Nanoparticles. Appl. Biochem. Microbiol..

[66] Godoy-Gallardo M., Eckhard U., Delgado L.M., de Roo Puente Y.J.D., Hoyos-Nogués M., Gil F.J., Perez R.A. (2021). Antibacterial Approaches in Tissue Engineering Using Metal Ions and Nanoparticles: From Mechanisms to Applications. Bioact. Mater..

[67] Flemming H.C., van Hullebusch E.D., Little B.J., Neu T.R., Nielsen P.H., Seviour T., Stoodley P., Wingender J., Wuertz S. (2025). Microbial Extracellular Polymeric Substances in the Environment, Technology and Medicine. Nat. Rev. Microbiol..

[68] Guo J., Qin S., Wei Y., Liu S., Peng H., Li Q., Luo L., Lv M. (2019). Silver Nanoparticles Exert Concentration-Dependent Influences on Biofilm Development and Architecture. Cell Prolif..

[69] Ghanaim A.M., Mohamed H.I., El-Ansary A.E. (2025). Production and Characterization of Exopolysaccharides from Pseudomonas Aeruginosa AG01 with Some Medical Potential Applications. Microb. Cell Fact..

